# Health Insurance

**Published:** 1920-07-10

**Authors:** 


### Health Insurance.

### New and Prospective Benefits.

The recent legislation effecting changes in National Health Insurance has come into force this week. Contributions have been raised, and there are to be material increases in benefits. The contributions are, roughly, 25 per cent. higher, but the benefits are about 50 per cent. higher. This tendency may be still further emphasised when the valuation, now in progress, of the approved societies is disclosed. It is forecasted that the valuations may produce surpluses, and that a large proportion of societies will be able to give additional benefits such as medical treatment for the wife and family of the insured, convalescent homes for members, or further cash benefits for sickness, disablement and maternity.

Under the present new scale the amount payable to a man who is away from his work through illness will be 15s. a week, instead of 10s. as formerly; to a woman worker, 12s. a week instead of the present 7s. 6d. a week. Disablement benefit will be 7s. 6d. a week in each case instead of 5s., and maternity benefit £2 instead of 30s. Sickness benefit lasts for twenty-six weeks in any one year, and after that it comes under disablement benefit, which is really a continuation of sickness benefit. Disablement benefit will cease at the age of seventy. In regard to maternity benefit, an employed married woman will receive £4, £2 being in respect of her own insurance, and £2 in respect of her husband's.

Steps are being taken by the Ministry of Health to improve the standard of medical benefit concurrently with the increased cash benefits. In future a limit will be placed on the number of insured persons which can be placed on a doctor's panel, the maximum in the case of a single-handed practitioner being 3,000. It is also provided that the local Insurance Committee may fix the maximum in any particular area at a lower figure. Moreover, patients will be able to change their

**THE PUBLIC WELL-BEING**—(*continued*).

doctor at the end of any half-year, instead of only at the end of the year. Another useful provision is that under which the nearest insurance practitioner is bound to give attention in case of accident or other sudden emergency, even though the insured person is not on his panel. The new Act also requires for the first time that a practitioner must provide sufficient surgery and waiting-room accommodation for his patients, having regard to their circumstances. Medical officers are about to be appointed by the Ministry with a view to improving the conditions of diagnosis and the treatment of disease among the workers. These officers may co-operate with insurance practitioners generally. The Ministry hope to have part of this scheme in operation in the next two or three months.

